# Anhedonia: Current and future treatments

**DOI:** 10.1002/pcn5.70088

**Published:** 2025-03-23

**Authors:** Alessandro Serretti

**Affiliations:** ^1^ Department of Medicine and Surgery Kore University of Enna Enna Italy; ^2^ Oasi Research Institute‐IRCCS Troina Italy

**Keywords:** anhedonia, antidepressants, major depressive disorder, psychopharmacology

## Abstract

Anhedonia is a transdiagnostic domain that leads to poor disorder outcome and low remission rates. This narrative review describes a broad range of interventions targeting anhedonia, including pharmacological, neuromodulatory, behavioral, and lifestyle‐based approaches. Drugs such as vortioxetine, agomelatine, bupropion, ketamine, and brexpiprazole show promising anti‐anhedonic effects, while traditional antidepressants, such as serotonin‐norepinephrine reuptake inhibitors (SNRIs) and, even more so, selective serotonin reuptake inhibitors (SSRIs), are less effective. Neuromodulation techniques, including repetitive transcranial magnetic stimulation, transcranial direct current stimulation, and transcutaneous auricular vagus nerve stimulation, proved effective at improving anhedonia, particularly when used in targeted areas. Psychotherapeutic interventions, including behavioral activation, mindfulness‐based strategies, and savoring techniques, also help re‐engage patients with pleasurable activities and enhance positive affect. Innovative treatments, such as aticaprant and psilocybin, showed promising results. Substantial evidence suggests that improving anhedonia leads to better psychosocial functioning, quality of life, and sustained remission. Although most data come from short‐term studies, several long‐term analyses suggest that maintaining hedonic improvements is feasible and beneficial. The reviewed evidence underscores the importance of routine assessment of anhedonia and the integration of symptom‐specific strategies. Tailoring interventions to address individual patterns of reward disruption may optimize outcomes for patients with anhedonia.

## Graphical abstract

Anhedonia is an independent transdiagnostic domain Traditional antidepressants may be partially effective. A number of pharmacological and nonpharmacological treatments are appropriate for treating anhedonia.

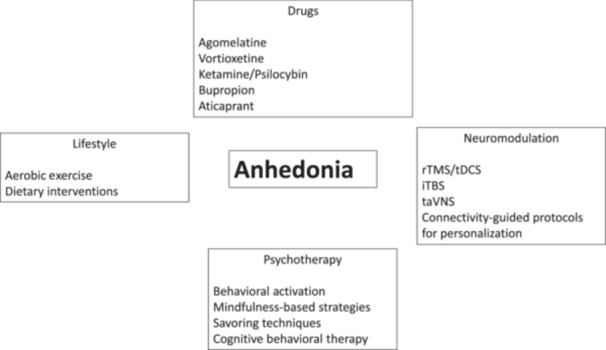

## INTRODUCTION

Anhedonia is defined as the diminished ability to desire and experience pleasure from activities that are typically enjoyable. The condition is increasingly recognized as an independent disorder domain across multiple psychiatric conditions (including major depression, schizophrenia, and substance use disorders) that independently predicts poorer outcomes, reduced quality of life, and higher rates of relapse.^1^, ^2^, ^3^, ^4^, ^5^, ^6^, ^7^, ^8^, ^9^, ^10^ This recognition stems from a growing body of research that highlights anhedonia not merely as a symptom but as a core and possibly independent domain that can significantly impact motivation, decision‐making processes, and overall quality of life. More in detail, anhedonia dimensions include reward components such as interest, motivation, effort expenditure, valuation, reward anticipation, learning, pleasure, and satiation. These dimensions are^1^, ^2^ usually grouped in anticipatory anhedonia, that is the reduced ability to experience pleasure in anticipation of rewarding events, and consummatory anhedonia, that refers to a reduced ability to experience pleasure from activities.^3^, ^11^ Furthermore, anhedonia has been linked to poorer treatment outcomes and increased risk of suicide, emphasizing the need for targeted interventions.^6^, ^12^, ^13^, ^14^

In recent years, both clinical research and practice have therefore begun to move beyond a narrow focus on mood, exploring the neurobiological, cognitive, and behavioral underpinnings of anhedonia. Functional imaging studies have identified key nodes in the brain's reward circuitry, including the ventral striatum, nucleus accumbens, and prefrontal cortex, that are implicated in various aspects of anticipatory and consummatory pleasure. Neurochemical findings point toward dopaminergic, glutamatergic, and serotonergic alterations in reward pathways, reinforcing the idea that therapies must be tailored to enhance positive affect, motivation, and reward responsiveness.^2^, ^6^, ^15^, ^16^, ^17^, ^18^, ^19^, ^20^ Additionally, genetic studies have begun to uncover specific genetic variants and heritable factors that may predispose individuals to anhedonic symptoms, indicating a specific biological underpinning to the disorder.^17^, ^21^, ^22^

This changing perspective is prompting a new wave of research into treatments that directly target these underlying mechanisms, ranging from novel pharmacological agents to neuromodulation devices to specialized psychotherapeutic interventions focused on reengaging patients with hedonic behaviors.

Pharmacotherapy, which has historically been based on serotonergic and noradrenergic modulation, is increasingly questioned for its variable effects on anhedonia. Traditional antidepressants often reduce overall depression severity yet may leave patients with residual anhedonia, thereby limiting their recovery. Novel treatments, including ketamine and related compounds, partial agonists at dopamine D2 and D3 receptors, and multimodal antidepressants, such as vortioxetine, demonstrate the potential to improve anhedonia more robustly.^6^, ^7^ Alongside pharmacological approaches, the role of inflammation, stress, and neurotrophic factors, such as brain‐derived neurotrophic factor (BDNF), have gained interest.^9^

Beyond medications, neuromodulation techniques have shown promise. Repetitive transcranial magnetic stimulation (rTMS), transcranial direct current stimulation (tDCS), intermittent theta‐burst stimulation (iTBS), and transcutaneous auricular vagus nerve stimulation (taVNS) are increasingly studied as ways to target and recalibrate dysfunctional reward‐related neural networks,^23^, ^24^, ^25^ particularly when guided by functional connectivity patterns to tailor stimulation.^23^, ^24^, ^25^

In a complementary way, psychotherapeutic interventions have also been investigated. Behavioral activation (BA) therapies, mindfulness‐based cognitive therapies, and savoring techniques aim to reinstate or strengthen reward responsiveness.^26^, ^27^ Similarly, lifestyle factors, including exercise and dietary modifications, may exert subtle but meaningful influences on anhedonia, although their efficacy can vary based on individual patient characteristics and baseline conditions.

Clinicians should therefore recognize and measure anhedonia routinely and consider it a core dimension of treatment target, complementing traditional depression rating scales. Measures such as the Snaith–Hamilton Pleasure Scale (SHAPS), the Temporal Experience of Pleasure Scale (TEPS), or anhedonia subfactors derived from established scales like the montgomery‐asberg depression rating scale (MADRS) can guide clinical decision‐making and tailor interventions to the needs of individual patients.

As previously mentioned, anhedonia is not unique to depression. Conditions like schizophrenia, addiction, and chronic pain states also show reward processing deficits. Thus, treatments validated for depression‐related anhedonia may have transdiagnostic utility. For example, improving hedonic capacity can help patients with schizophrenia address persistent negative symptoms like social withdrawal and reduced motivation.^28^ Similarly, interventions that restore normal reward system functioning can decrease cravings and drug‐seeking behavior in substance use disorders, suggesting a shared pathophysiological substrate where anhedonia acts as a crucial point.

Some challenges still remain. The heterogeneity of anhedonia (e.g., reduced anticipation versus reduced enjoyment, or deficits in motivation versus deficits in consummation) requires a specific understanding of which components of reward processing are impaired in each patient, though frequently they co‐occur. Personalized approaches, supported by biomarkers such as brain‐imaging findings or inflammatory markers, may allow clinicians in the near future to choose the most suitable treatments.^29^

The aim of this narrative review is to integrate and examine a wide array of representative studies that have explored the treatment of anhedonia, spanning from pharmacological interventions, neuromodulation, psychotherapy, to lifestyle modifications.

## METHODS

A comprehensive and nonsystematic survey of the recent literature on the treatment of anhedonia was conducted. Study selection focused mainly on major depressive disorder (MDD) but other disorders were included when related to anhedonia. The review followed a flexible approach designed to capture a range of research directions and conceptual advances across multiple domains and conditions based on previous reviews and current literature.^6^, ^7^, ^9^

Intervention modalities of interest included traditional antidepressants, novel pharmacological agents, behavioral and mindfulness‐based psychotherapies, neuromodulation techniques, non‐pharmacological lifestyle interventions, and emerging modalities, such as psychedelic‐assisted therapy. Iterative searches were performed in electronic databases—principally PubMed and Google Scholar—focusing on research published primarily in the past two decades. Search terms included “anhedonia,” “reward processing,” “treatment,” “intervention,” “depression,” “ketamine,” “rTMS,” “CBT,” “behavioral activation,” and “vortioxetine” among others in various combinations. Studies cited within identified articles and relevant reviews, as well as important historical and conceptual papers that provided context on the evolution of the anhedonia construct and its clinical relevance were also included.

Peer‐reviewed original research articles, randomized controlled trials, post‐hoc analyses, and open‐label studies that reported on treatments specifically targeting or measuring changes in anhedonia were prioritized. Narrative and systematic reviews were considered to ensure coverage of established findings and consensus views, as well as mechanistic studies that linked clinical improvements to neurobiological or behavioral endpoints. While the majority of included references were human studies, preclinical work was considered when it offered valuable mechanistic insights.

## RESULTS

### Agomelatine

Agomelatine's unique mechanism—as a melatonergic agonist and serotonergic antagonist—makes it promising for treating anhedonia.

An early study,^30^ conducted an 8‐week open‐label study involving 30 patients with MDD to evaluate the impact of agomelatine (25–50 mg daily) on depression, anxiety, anhedonia, and sleep quality; they found significant improvements in Hamilton Depression Rating Scale (HAM‐D) scores (*p* < 0.05), Hamilton Anxiety Rating Scale (HAM‐A) scores (*p* < 0.01), Snaith–Hamilton Pleasure Scale (SHAPS) scores (*p* < 0.05), and Leeds Sleep Evaluation Questionnaire (LSEQ) scores (*p* < 0.05), with 60% achieving remission and no serious adverse events reported, thus demonstrating early clinical benefits and particularly underscoring agomelatine's positive effects on anhedonia. In a subsequent 8‐week open‐label, parallel‐group pilot study, the same group^31^ compared agomelatine (25–50 mg/day) and venlafaxine XR (75–150 mg/day) in 60 outpatients with MDD; while both treatments reduced depressive (HAM‐D) and anxiety (HAM‐A) symptoms, agomelatine demonstrated a significantly greater reduction in anhedonia (SHAPS) (*F* = 20.74, *p* < 0.001) than venlafaxine (*F* = 3.27, *p* < 0.05), with improvements evident as early as Week 1 in the agomelatine group. Additionally, clinical global impressions scale (CGI) was significant in the agomelatine arm but not with venlafaxine, and agomelatine had fewer adverse events and better tolerability. Several years later, an 8‐week open‐label, multicenter, real‐world Phase IV study^32^ enrolled 257 outpatients with a major depressive episode and analyzed data from 143 patients treated with agomelatine 25–50 mg; they reported significant improvements in SHAPS from a baseline of 8.5 to 4.1 at Week 8 (*p* < 0.001), with notable improvements occurring even by Week 1 (*p* < 0.01). Depression severity (quick inventory of depressive symptomatology, self‐report‐SR‐16]) and anxiety (general anxiety disorder‐7) scores also improved significantly (*p* < 0.001), with 65.7% achieving response and 49.6% reaching remission, and positive correlations emerged between changes in SHAPS and other symptom scales. A further study by Martinotti et al.^33^ focused on biological correlates by conducting an 8‐week open‐label study of 27 patients with depressive disorders spectrum to assess the impact of agomelatine (25–50 mg/day) on serum BDNF levels and their correlation with clinical improvements. Results showed a significant increase in BDNF at Week 2 and at Week 8, accompanied by decreases in HDRS, HARS, and SHAPS scores. Correlations between BDNF changes and improvements in depression and anhedonia supported the notion that biological markers like BDNF may underlie the mechanistic action of agomelatine on mood and hedonic capacity. A larger‐cohort study of 1570 outpatients with MDD treated by general practitioners with agomelatine assessed outcomes at baseline and after 10–14 weeks.^34^ Using MADRS for depression severity, questionnaire for social functioning for psychosocial functioning, SHAPS for anhedonia, and CGI for global assessment, they reported that improvement in anhedonia was the strongest predictor of psychosocial improvement with an impressive odds ratio of 7.3. Mediation analyses confirmed that improvements in depressive symptoms influenced psychosocial functioning primarily through changes in anhedonia, indicating that persistent anhedonia might underlie residual functional impairment despite clinical remission of other depressive symptoms.

With a focus on inflammatory aspects, a small study was conducted on 30 MDD outpatients to explore the possible effects of agomelatine on C‐reactive protein (CRP) levels.^35^ They reported a reduction in HAM‐D and SHAPS scores alongside a significant decrease in CRP. Interestingly, remitters showed greater CRP reductions, and CRP variation correlated with baseline depression severity, supporting the contribution of an inflammatory component to treatment response and the possibility that agomelatine's improvement in anhedonia could be linked, at least in part, to modulating inflammatory pathways. The same group of the original 2011 study performed a pooled analysis of data from two international, open‐label, noninterventional studies conducted between 2011 and 2015 at 353 centers across seven countries, including a total of 1942 MDD patients treated with agomelatine for up to 12 weeks.^36^ They reported a significant decrease in short‐version montgomery‐asberg depression rating scale scores with 79.2% achieving response, 63.9% remission, and 27.9% complete remission. SHAPS scores improved markedly with 84.8% of patients achieving anhedonia remission (SHAPS ≤ 2). Predictors of better outcomes included baseline severity, changes in anhedonia, and the absence of concomitant treatment, while agomelatine was generally well tolerated with a low rate of adverse events (2.4%) and no serious events reported.

An interesting comparison study conducted a 24‐week randomized controlled trial comparing agomelatine (25–50 mg/day) and escitalopram (10–20 mg/day) in 324 patients with moderate‐to‐severe MDD.^37^ Both treatments yielded significant antidepressant efficacy, but agomelatine showed superior improvements in patients with pronounced sleep complaints and was associated with less emotional blunting than escitalopram, evidenced by fewer reports of reduced emotional intensity and reduced interest in previously cared‐about things. While this study focused on sleep and emotional experiences, the reduced blunting with agomelatine indirectly hinted at more robust preservation or restoration of hedonic responsiveness compared to selective serotonin reuptake inhibitors (SSRIs), as we will see in a following section.

Finally, the most recent study added a novel component by examining the combined effect of agomelatine and aerobic exercise (AE) on moderate to severe depression.^38^ In this 12‐week randomized trial, 178 patients were allocated to receive either agomelatine alone or agomelatine plus AE. Both groups displayed significant decreases in HAM‐D, Beck's depression inventory (BDI), and SHAPS scores, but the combination group achieved greater improvements and significantly reduced CRP levels compared to agomelatine alone. Clinically, 34 patients (38.64%) achieved remission in the AG + AE group versus 27 (30%) in the AG‐alone group, and the combination group also experienced fewer adverse events.

Taken together, these studies support the capacity of agomelatine to improve not only depressive and anxiety symptoms but also hedonic tone and sleep quality. Biological studies that measured biomarkers such as BDNF and CRP levels suggested that the mechanisms underpinning agomelatine's anti‐anhedonic and antidepressant effects may be linked to neurotrophic and anti‐inflammatory pathways.

### Bupropion monotherapy and adjunctive dextromethorphan

Bupropion, a norepinephrine‐dopamine reuptake inhibitor (NDRI), has been extensively studied for its role in improving anhedonia, particularly through its dopaminergic and noradrenergic mechanisms.

The earlier study conducted a retrospective review of 27 outpatients with MDD who had shown only partial or unsatisfactory responses to either a serotonin reuptake inhibitor (SSRI) or bupropion monotherapy, examining the effects of adding bupropion to an ongoing SSRI or vice versa.^39^ Patients were treated for a relatively long time, mean of 11 ± 14 months, with average doses of bupropion at 243 ± 99 mg/day and SSRIs at fluoxetine‐equivalents of 31 ± 16 mg/day. Of the 27 individuals, 19 (70%) exhibited greater symptomatic improvement under the combined treatment than they had under either monotherapy. Although the data were uncontrolled and retrospective, the findings suggested that bupropion combination therapy might enhance treatment response in patients who do not fully improve with a single SSRI agent. Several years later, a more rigorously controlled randomized, double‐blind, placebo‐controlled study focused on bupropion sustained‐release (SR) and its effects on specific mood dimensions, particularly anhedonia.^40^ This investigation included a small sample of 19 outpatients with recurrent MDD who were assigned either to bupropion SR (*n* = 10) or placebo (*n* = 9) during a 6‐week double‐blind phase (Phase 1), followed by a 6‐week open‐label phase (Phase 2) in which placebo patients were switched to bupropion, and bupropion patients had their dose increased from 300 to 400 mg/day. Bupropion SR achieved a significant linear decline in anhedonic scores compared to placebo during Phase 1. While anxiety measures (general distress anxiety, anxious arousal) and depression severity (HAM‐D‐17) improved over time in both groups, the effects on anhedonia were more distinctly pronounced with bupropion, and these became more evident at higher dosing in Phase 2.

A later study focused on early effects, investigating the acute effects of a single dose of bupropion SR (150 mg) on emotional and reward processing in 40 healthy volunteers with no psychiatric diagnoses. In a randomized, double‐blind, placebo‐controlled design, participants were tested at 3 h.^41^ Bupropion significantly improved the recognition of ambiguous facial expressions as happy and reduced attention to fearful faces, indicating an early shift in emotional bias. It also reduced false recognition of negative words, suggesting a decrease in negative cognitive biases. Intriguingly, despite these positive changes in emotional processing, bupropion failed to enhance reward processing. This acute dissociation—improved positive emotional processing alongside diminished reward sensitivity—highlighted that early bupropion effects may differ from its longer‐term outcomes in clinical samples. Subjective measures (SHAPS) increased in the bupropion group at a trend level, suggesting a possible early adverse effect on hedonic capacity. A more recent study investigated the dextromethorphan and bupropion combination, which was recently approved by the US Food and Drug Administration.^42^ This Phase 2, randomized, double‐blind, active‐controlled trial examined AXS‐05—oral tablet combining dextromethorphan and bupropion—against bupropion SR alone in patients with moderate or greater severity MDD. Eighty patients completed efficacy assessments and treatment lasted 6 weeks. On the primary outcome measure, the MADRS total score change from baseline, AXS‐05 was superior to bupropion. This advantage emerged as early as Week 2 and persisted through Week 6. Secondary outcomes confirmed this superiority: Remission rates at Week 6 were significantly higher in the AXS‐05 group (46.5%) compared to bupropion (16.2%), and improvements on the MADRS‐6 Core Symptom subscale were also significantly greater with AXS‐05. Notably, AXS‐05's enhanced efficacy suggests a synergistic effect between the NMDA receptor antagonist/σ‐1 receptor agonist properties of dextromethorphan and the dopaminergic/noradrenergic reuptake inhibition provided by bupropion. Safety profiles were generally favorable, with common adverse events including dizziness, nausea, and dry mouth. Unlike standard bupropion, the combined agent showed rapid and robust antidepressant effects, presumably strengthening both affective and reward‐related symptom domains, though this study did not break down results specifically for anhedonia metrics. However, given that anhedonia and core depressive symptoms improved more with AXS‐05, it may be inferred that the augmentation of bupropion's pharmacological activity by dextromethorphan could contribute to more pronounced and earlier improvements in hedonic functioning.

Taken together, these studies suggest that bupropion, through its dopaminergic and noradrenergic mechanism, appears particularly relevant for addressing anhedonia.

### Selective serotonin reuptake inhibitors and serotonin‐norepinephrine reuptake inhibitors

Selective serotonin reuptake inhibitors (SSRIs) and serotonin‐norepinephrine reuptake inhibitors (SNRIs) are among the most widely prescribed classes of antidepressants for MDD. Despite their efficacy in depression, their impact on anhedonia is less clear. The evidence from clinical studies suggests a mixed profile for SSRIs and SNRIs, with concerns regarding the potential emotional blunting effects and diminished reward sensitivity contrasting with their broader antidepressant effects.^43^, ^44^

A relatively old but interesting study provides insights into the temporal pattern of symptom improvement in MDD by showing that, in an open‐label study involving 140 patients treated with sertraline (50–150 mg/day) for 8 weeks, anxiety symptoms improved first (Days 0–7), followed by depressive symptoms (Days 7–21), and finally anhedonia (Days 21–56), based on clinician‐rated and patient‐rated symptom clusters.^45^ These findings suggested that while anxiety and core depressive features respond relatively quickly to antidepressant treatment, anhedonia may take longer to improve. More recently, a study investigated the neural underpinnings of reward and aversion processing in a double‐blind, parallel‐group fMRI study of 45 healthy participants receiving 7 days of either citalopram, reboxetine, or placebo.^46^ Although no subjective changes in pleasantness or wanting were reported, citalopram diminished ventral striatal and orbitofrontal cortex responses to rewarding chocolate stimuli and similarly reduced responses to aversive stimuli, whereas reboxetine enhanced medial orbitofrontal cortex activation to chocolate. This neural evidence provided an early mechanistic explanation for the emotional blunting associated with SSRIs and underscored the importance of differentiating serotonergic and noradrenergic mechanisms when targeting anhedonia. A related neuroimaging study focusing on positive emotion regulation^47^ recruited 27 medication‐free adults with MDD and 19 healthy controls to examine right ventrolateral prefrontal cortex (RVLPFC) activity during suppression of positive affect. Lower RVLPFC activity when trying to suppress positive emotions predicted greater reductions in anhedonia (as measured by the mood and anxiety symptom questionnaire anhedonic depression [MASQ‐AD]) after 8 weeks of venlafaxine‐ER or fluoxetine treatment, explaining 61% of the variance in anhedonia improvement. This finding suggested that recovery from anhedonia may be more difficult in patients who have an excessive, and automatic, top‐down inhibition of positive affect. There are also implications for the direction of concomitant psychotherapies, as the authors suggest anhedonic patients may also need training in how not to suppress the experience of positive emotions once they are in contact with positive stimuli. Moreover, the findings may also suggest a potential neural marker for treatment response.

Levomilnacipran is an SNRI that is not available in Europe, but it is approved by the US Food and Drug Administration. A post‐hoc analysis of five randomized, double‐blind, placebo‐controlled trials evaluated levomilnacipran ER in 2598 patients with MDD.^48^ The analysis indicated that levomilnacipran ER significantly improved individual MADRS items and symptom clusters, with particularly notable improvements in anhedonia and related domains as well as retardation and dysphoria. These improvements and higher odds relative to placebo reinforced the idea that targeting both serotonin and norepinephrine might be beneficial for core motivational deficits.

Another study explored the sensory dimension of hedonic processing by examining changes in olfactory hedonic perception in a sample of 43 patients undergoing an 8‐week open‐label escitalopram trial.^49^ Only responders, defined as those with ≥50% reduction in MADRS scores, showed significant improvements in the hedonic perception of pleasant odors after treatment, while nonresponders did not. No changes were noted in olfactory threshold or identification, nor in the perception of unpleasant odors. This study highlighted a novel sensory marker—hedonic odor perception—as potentially reflective of underlying changes in emotional and motivational circuits associated with successful treatment of MDD. Finally, a 2024 post‐hoc pooled analysis focused on venlafaxine XR's efficacy in improving anhedonia and amotivation.^50^ In a sample of 1087 participants drawn from five randomized, placebo‐controlled trials, venlafaxine XR significantly reduced MADRS‐derived anhedonia scores compared to placebo, with differences emerging as early as Week 2 and becoming more pronounced by Week 8. Similarly, amotivation improved faster and more robustly in the venlafaxine XR group. Patients with more severe baseline anhedonia or amotivation benefited most.

In conclusion, SSRIs alone did not show a particularly relevant effect on anhedonia, probably because of their emotional blunting effects, but SNRIs are more promising and combination treatments may further improve efficacy on anhedonia.

### Ketamine

Ketamine and esketamine are emerging as promising treatments for MDD and possibly for anhedonia. Ketamine, a rapid‐acting NMDA receptor antagonist, produces its effects through distinct pathways that converge on neural circuits responsible for reward anticipation, motivation, and pleasure.^51^

One of the earlier studies, a randomized, double‐blind, placebo‐controlled, crossover study, involved 36 patients with treatment‐resistant bipolar depression to investigate ketamine's anti‐anhedonic effects and their neural correlates.^52^ Participants received a single intravenous infusion of ketamine (0.5 mg/kg) and a placebo infusion, separated by a 2‐week washout. Ketamine significantly reduced SHAPS scores compared to placebo, with effects evident within 40 min and persisting up to 14 days. These improvements in anhedonia remained significant even after controlling for overall depressive symptom reduction. Positron emission tomography (PET) scans showed that increases in dorsal anterior cingulate cortex (dACC) and right putamen glucose metabolism correlated with the anti‐anhedonic response. A similar study examined ketamine's effects in 52 patients with treatment‐refractory MDD receiving a single infusion of ketamine (0.5 mg/kg) in an open‐label design, with 20 patients undergoing 2‐[(18)F]fluoro‐2‐deoxy‐D‐glucose positron emission tomography before and 2 h after infusion.^53^ Ketamine improved SHAPS scores as early as 40 min post‐infusion, with effects lasting up to 3 days. Changes in hippocampus and dACC glucose metabolism correlated with reduced anhedonia, and decreases in orbitofrontal cortex metabolism were also observed. Notably, these neural changes were specific to anhedonia and persisted after controlling for global depressive symptoms.

From a purely clinical perspective, a post‐hoc analysis of data from 100 participants (65 with MDD, 35 with bipolar disorder) was performed in three clinical ketamine trials with focus on suicidal ideation.^54^ Baseline anhedonia (SHAPS scores) was significantly associated with suicidal ideation even after controlling for overall depressive severity. One day after ketamine infusion, reductions in SHAPS scores remained significantly correlated with decreases in suicidal ideation independent of changes in general depressive symptoms, highlighting the clinical relevance of targeting anhedonia to reduce suicide risk.

A systematic review on 30 studies (11 clinical, 19 preclinical) investigated ketamine's effect on anticipatory, consummatory, and motivation‐related reward deficits.^55^ Across human studies, all eight that used the SHAPS reported significant reductions in consummatory anhedonia following ketamine treatment. Preclinical data supported improvements in anticipatory and motivation‐related deficits, linked to enhanced BDNF signaling, glutamate release, and improved dopaminergic function. These findings underscored ketamine's broad spectrum of anti‐anhedonic effects. More recently, a double‐blind, placebo‐controlled, crossover study investigated ketamine's effects on anterior cingulate cortex (ACC) resting‐state functional connectivity (rsFC) and their relationship with anhedonia in treatment‐resistant depression.^56^ Fifty participants were included (29 with treatment‐resistant depression, 21 healthy controls), and rsFC was measured via resting‐state fMRI 2 days post‐infusion. Ketamine significantly improved depressive symptoms and improved anticipatory and consummatory pleasure measured by the TEPS. SHAPS scores showed a trend‐level improvement. Increased subgenual ACC connectivity to the perigenual ACC, ventral striatum, and anterior ventromedial prefrontal cortex correlated with improvements in SHAPS scores and TEPS‐Anticipatory scores.

Taken together, the evidence strongly suggests that novel rapid‐acting treatments like ketamine and esketamine exert potent anti‐anhedonic effects, affecting distinct yet interconnected neural circuits involved in reward processing.

### Psilocybin

Psilocybin, a serotonergic psychedelic targeting 5‐HT2A receptors, is also a promising potential treatment for anhedonia. An open‐label feasibility study in 12 patients was performed in treatment‐resistant unipolar major depression.^57^ Two doses of psilocybin (10 mg followed by 25 mg 1 week later) combined with psychological support led to significant reductions in depression and anhedonia. SHAPS scores decreased (Hedges' *g* = 2.7), and improvements persisted for up to 3 months. This suggested that rapid and enduring reductions in anhedonia can occur with psilocybin.

### Vortioxetine

Vortioxetine, a multimodal antidepressant with a unique mechanism targeting 5‐HT receptors and the serotonin transporter, has been evaluated extensively for its role in improving anhedonia in MDD and related conditions.

Cao et al.^58^ provided some of the earliest evidence that vortioxetine can specifically improve anhedonia in MDD beyond its effects on overall depressive symptoms. In this open‐label, 8‐week study of 95 adults with MDD, vortioxetine (10–20 mg/day) significantly reduced anhedonia as measured by the SHAPS and the MADRS anhedonia factor. Improvements in anhedonia correlated with enhanced social functioning (Sheehan Disability Scale) and quality of life (WHO‐5), and a mediation analysis showed that reductions in anhedonia explained a substantial portion (nearly 40%) of the variance linking overall symptom improvement to better social functioning. Building on this foundational evidence, a large post‐hoc pooled analysis investigated 11 short‐term, placebo‐controlled trials encompassing 3219 patients treated with vortioxetine (5–20 mg/day) and 1769 placebo recipients.^59^ Their results indicated that vortioxetine robustly improved anhedonia in a dose‐dependent manner: for example, the MADRS Anhedonia subscale improved by −0.97 points more than placebo at 5 mg/day (*p* = 0.009) and by −2.24 points at 20 mg/day (*p* < 0.001). These anhedonia improvements also mediated enhancements in patients' functioning, underscoring that targeting anhedonia is central to restoring patients' quality of life. Expanding the treatment target, another study explored vortioxetine's cognitive benefits in a sample of 83 adults with mild cognitive impairment but without significant depression (patient health questionnaire‐9 ≤ 4).^60^ Over 6 months of open‐label treatment (5–10 mg/day), participants' cognition improved substantially, and while this study did not specifically measure anhedonia, the cognitive gains align with vortioxetine's multimodal profile and suggest its potential to address complex neuropsychiatric features often intertwined with reward processing.

Findings may extend also to Asian populations. A post‐hoc analysis of an 8‐week Japanese Phase 3 trial compared vortioxetine (10 and 20 mg/day) to placebo in 489 patients with MDD and high baseline severity (MADRS ≥ 26).^61^ Both vortioxetine doses significantly reduced the MADRS anhedonia factor score compared to placebo, with a larger effect of the 20 mg dose, with more pronounced improvements observed in patients presenting higher baseline anhedonia.

A recent long‐term analysis was reported on vortioxetine from two open‐label, 52‐week extension studies that followed prior double‐blind trials.^62^ In total, 74 participants continued at 5–10 mg/day and 71 at 15–20 mg/day. Over the entire year, vortioxetine was well‐tolerated and sustained or further improved baseline gains achieved in short‐term phases. Crucially, a post‐hoc mixed model for repeated‐measures analysis revealed sustained and significant improvements in the MADRS anhedonia factor, with reductions of 3.10 ± 0.57 points in the lower‐dose cohort and 5.62 ± 0.60 points in the higher‐dose cohort, confirming that vortioxetine not only maintains but may increase its anti‐anhedonic effect over long‐term treatment. Finally, a recent study extended vortioxetine's profile into a different diagnostic population—patients with remitted schizophrenia.^63^ This randomized trial of 120 patients, half receiving vortioxetine 10 mg/day added to their stable antipsychotic regimen and half continuing antipsychotic monotherapy, measured anhedonia using the Chapman Physical Anhedonia Scale and Chapman Social Anhedonia Scale. Over 12 weeks, vortioxetine significantly reduced both physical and social anhedonia, while control patients saw no improvement. The main effect sizes were large (*η*² = 0.733 for physical and *η*² = 0.618 for social anhedonia), and the largest improvements were observed among patients receiving vortioxetine combined with olanzapine.

Taken together, this body of research consistently demonstrates vortioxetine's capacity to target anhedonia transdiagnostically. The drug's capacity to improve anhedonia appears to be dose‐dependent, long‐lasting, associated with improvements in functioning and quality of life, and generalizable across different populations (from MDD patients with various severity levels to individuals with schizophrenia and mild cognitive impairment).

### Psychotherapies

Psychotherapy is traditionally considered complementary in the treatment of MDD and a series of recent studies support a possible role also in anhedonia.

Garland et al.^64^ provided evidence that mindfulness‐based interventions can meaningfully impact anhedonia by enhancing neurophysiological responses to natural rewards, as demonstrated in a sample of 63 veterans with chronic pain on long‐term opioid therapy (LTOT). In that study, participants randomized to mindfulness‐oriented recovery enhancement (MORE) showed significant reductions in SHAPS scores at follow‐up relative to a supportive group, bringing anhedonia closer to normal levels. These decreases correlated with increased late positive potentials (LPPs) and skin conductance levels in response to natural reward cues, with LPP changes during savoring predicting reductions in anhedonia.

Other approaches have been investigated as well. A secondary analysis was performed on cognitive behavioral therapy (CBT) and behavioral activation (BA) effects on anhedonia in MDD.^65^ While both interventions significantly reduced SHAPS scores from baseline to 6 months, improvements plateaued thereafter and never reached normative, low‐anhedonia levels. Furthermore, baseline anhedonia predicted poorer long‐term depression outcomes, particularly in BA.

A comparison study compared 12 weeks of CBT with escitalopram (10–20 mg/day) in 26 participants with MDD, using measures like the HAM‐D, BDI, SHAPS, and various cognitive assessments.^66^ Both treatments improved depressive symptoms, anhedonia, and maladaptive cognitions equally, suggesting that, despite differing modalities, CBT and escitalopram may achieve comparable changes in the cognitive and hedonic dimensions of MDD.

Given the limitations of in‐person psychotherapies, Internet‐based CBT (iCBT) effects on reward circuit functioning in MDD were investigated.^67^ Among 94 participants (26 in iCBT, 26 in monitored attention control, and 42 healthy controls), iCBT led to significant reductions in SHAPS scores and enhanced nucleus accumbens (Nacc) and subgenual ACC activation during the Monetary Incentive Delay task. Mediation analyses indicated that increased Nacc activity accounted for anhedonia reductions.

A further approach was investigated in a randomized controlled trial examining Future Event Specificity Training (FEST) on undergraduates.^68^ FEST improved episodic future thinking metrics—such as specificity, detail, and perceived control—but had no significant effect on anhedonia or dampening of positive emotions. However, this may be due to baseline mild symptom severity.

Behavioral activation is also receiving increased interest. A randomized trial compared behavioral activation treatment for anhedonia (BATA) and mindfulness‐based cognitive therapy (MBCT) in 73 transdiagnostic subjects.^69^ While both treatments reduced SHAPS scores over time, no between‐condition differences emerged. Critically, within‐person homework completion predicted greater session‐to‐session improvements in anhedonia, particularly in BATA when clinician‐reported homework was considered. These results emphasize the importance of patient engagement and session‐level processes for optimizing therapeutic outcomes. In a follow‐up study, a larger parallel‐arm randomized control trial (RCT) compared BATA and MBCT for clinically significant anhedonia in a transdiagnostic cohort of adults with clinically significant anhedonia (SHAPS ≥ 20).^70^ Both treatments yielded substantial SHAPS reductions of ~7 points without significant differences between them. Similarly, internalizing symptoms decreased in both conditions. Notably, MBCT showed a trend toward higher attrition, suggesting that behavioral activation approaches may be slightly more tolerable for some individuals. A modified approach was investigated testing a brief behavioral activation plus savoring (BA + S) intervention against emotional awareness (EA) control in 60 university students with low positive affect.^71^ BA + S significantly improved daily positive affect and positive valence symptoms while also reducing negative valence symptoms more than control.

Potsch and Rief^72^ added another piece to the picture by testing 2‐week online interventions: behavioral activation (BA), mindfulness and gratitude (MG), their combination (COM), and a waitlist control. BA and MG alone improved reward sensitivity and reduced depressive symptoms more than waitlist, while all active interventions (BA, MG, COM) reduced anhedonia (SHAPS) significantly. Contrary to expectations, combining BA and MG did not produce additive benefits. Finally, Garland et al.^73^ examined MORE's efficacy in a larger sample (*n* = 230) of veterans and military personnel with chronic pain on LTOT. Compared to supportive psychotherapy, MORE significantly reduced anhedonia, increased positive affect, and lowered opioid dose. Additionally, it improved pain interference, pain severity, and craving while promoting savoring and mindful reinterpretation.

In conclusion, this body of evidence demonstrates that treatments explicitly targeting reward‐related processes and anhedonia—be they behavioral activation variants, mindfulness‐based interventions, CBT enhancements, or novel approaches like savoring and future event specificity—are promising tools for improving outcomes in populations with low positive affect, depression, and even chronic pain/opioid dependence.

However, population and setting appear relevant: Veterans on LTOT, students with low positive affect, chronic depression samples, and patients with post‐traumatic stress disorder or chronic anxiety all respond somewhat differently to interventions. Identifying moderators—such as baseline anhedonia severity, patient engagement, personality traits like conscientiousness, and hormonal or neurobiological markers—might enhance precision and improve response rates.

### Brain stimulation

Brain stimulation strategies are also receiving much interest in regards to depression and other conditions^74^ and have been evaluated for the treatment of anhedonia.

Pettorruso et al.^75^ provided some of the earliest evidence that noninvasive brain stimulation can improve anhedonia in a substance‐use context by delivering high‐frequency (15‐Hz) repetitive transcranial magnetic stimulation (rTMS) to the left dorsolateral prefrontal cortex (DLPFC) in 15 individuals with chronic cocaine use disorder. After 10 sessions administered over 5 days, participants showed significant increases in both anticipatory and consummatory pleasure and decreases in anhedonia‐related subscales of the Cocaine Selective Symptoms Assessment. These hedonic improvements were also strongly correlated with reductions in craving. The following year, another study by Light et al. investigated the effects of rTMS in MDD from a different angle, focusing on the Happy Faces task to measure subtle changes in behavioral anhedonia.^76^ In a sample of 19 patients with MDD, 20 sessions of left DLPFC rTMS improved both subjective anhedonia (SHAPS) and the ability to detect subtle happy facial expressions, indicating that rTMS not only reduces anhedonia but also enhances sensitivity to positive social cues, with empathic happiness changes mediating these effects.

Expanding to other conditions, Bodén et al.^25^ focused on negative symptoms—including anhedonia, avolition, and blunted affect—in patients with either schizophrenia spectrum disorders or depression. Their randomized, sham‐controlled trial applied intermittent theta‐burst stimulation (iTBS) over the dorsomedial prefrontal cortex (DMPFC) for 2 weeks. While the overall sample (*n* = 56) did not show significant differences between active and sham stimulation, a subgroup analysis revealed that depressed patients showed a significant reduction in Clinical Assessment Interview for Negative Symptoms total scores compared to sham, suggesting that DMPFC iTBS could be targeted as an example to schizoaffective subjects.

With a focus on treatment resistance, a large retrospective study was conducted on 144 patients with treatment‐resistant MDD receiving standard rTMS to the left DLPFC.^77^ Their results were striking: SHAPS scores decreased from a mean of 8.10 at baseline to 3.06 posttreatment, marking a 58% improvement in anhedonia. This robust effect was comparable to the observed reduction in depression severity and demonstrated that even patients with severe baseline anhedonia responded well to rTMS.

With a focus on anticipatory anhedonia, connectivity‐guided rTMS was used to target the DLPFC–nucleus accumbens network in depressed individuals.^78^ In the randomized, double‐blind, sham‐controlled trial (*n* = 56), active 10‐Hz rTMS significantly improved anticipatory pleasure and enhanced electrophysiological markers of reward processing (cue‐N2 and cue‐P3 amplitudes during the Monetary Incentive Delay task). Improvement in cue‐P3 correlated with TEPS changes, highlighting a direct link between neural indices of reward anticipation and clinical gains. The evidence for noninvasive neuromodulation in treating anhedonia is confirmed in a recent study testing transcranial direct current stimulation (tDCS) in 70 depressed patients.^24^ The randomized, double‐blind, sham‐controlled trial compared anodal tDCS over the left DLPFC, cathodal tDCS over the right orbitofrontal cortex (OFC), and sham. After 4 weeks of treatment, left DLPFC tDCS showed the most enduring effect on anhedonia (up to 85.99% reduction in SHAPS at 8 weeks), while OFC‐targeted tDCS provided moderate, though less pronounced, benefits.

A direct comparison between rTMS and antidepressants switch has been recently reported in 89 patients with moderately treatment‐resistant depression.^79^ The study found that rTMS was not only more effective in reducing overall depression severity but also showed superior improvements in anhedonia and anxiety. With response rates significantly higher in the rTMS group, this study suggested that when standard pharmacotherapy fails, rTMS could represent a strong alternative for addressing core symptom dimensions like anhedonia.

Trying again in negative symptomatology, a multicenter, placebo‐controlled trial in patients with schizophrenia examined whether iTBS or tDCS targeting the right DLPFC could alleviate apathy, a negative symptom overlapping with anhedonia in its motivational deficits.^80^ Among 88 patients, neither active iTBS nor tDCS outperformed sham in reducing apathy or related symptoms. This negative result confirms the previous evidence of a marginal effect in schizophrenia. Finally, Ferstl et al.^81^ explored a different noninvasive neuromodulation method—transcutaneous auricular vagus nerve stimulation (taVNS)—in MDD and healthy controls. In this single‐blind, randomized crossover design with 59 participants, taVNS rapidly enhanced effort‐related invigoration and reward‐wanting in depressed patients during a behavioral task involving effort for food or money rewards. Interestingly, healthy controls showed a taVNS‐induced willingness to work for less desired rewards (reduced utility slope), whereas MDD patients benefited primarily from increased wanting and motivation, suggesting condition‐specific effects on the reward system.

In conclusion, these studies suggest a varying effect of neuromodulation's role in treating anhedonia. No single intervention is universally efficacious. Carefully chosen targets and stimulation parameters—tailored to specific symptom profiles and diagnoses—hold great potential for meaningfully restoring hedonic capacity in patients for whom standard treatments have proven insufficient.

### Other treatments

In this section a series of other possible treatments are discussed. Given the benefits observed by bupropion, other dopaminergic drugs have also been tested. A study in a non‐psychiatric population examined the effects of the dopamine agonist pramipexole in 657 patients with Parkinson's disease (PD).^82^ In this open‐label study, patients received pramipexole (mean dose ~1.0 mg/day) as add‐on therapy to levodopa for an average of 9 weeks. At baseline, anhedonia was present in nearly half the cohort (45.7%), and depression was also common. Following pramipexole treatment, anhedonia, as measured by the SHAPS, significantly improved, with a decline in prevalence from 45.7% to 25.5% overall, and even more pronounced improvements in those with moderate‐to‐severe depression. Depression also significantly decreased, and motor symptoms improved, indicating pramipexole's potentially broad neuropsychiatric benefits.

A broader pharmacodynamic target benefit was also supported by a post‐hoc analysis of four randomized, double‐blind, placebo‐controlled Phase 3 studies investigating adjunctive brexpiprazole in patients with MDD who did not respond adequately to one to three antidepressant treatments.^83^ Among the MADRS‐derived symptom clusters examined, anhedonia showed the largest effect size improvements when brexpiprazole was added, with statistically significant differences versus placebo. Improvements appeared as early as Week 1 and persisted through Week 6.

More recently, Grillo et al.^84^ approached anhedonia from a metabolic and dietary perspective. Working with a rodent model that mimics metabolic syndrome via hypothalamic insulin receptor downregulation (hypo‐IRAS rats), they found that obesity‐induced anhedonia—operationalized via reduced sucrose preference—could be both prevented and reversed by implementing dietary restriction. In ad‐libitum‐fed hypo‐IRAS rats, sucrose preference declined compared to controls, and these rats also displayed altered metabolic and inflammatory markers, including elevated leptin, triglycerides, interleukin (IL)‐1α, IL‐6, and CRP. Restricting food intake starting either early (Prevention group) or later (Reversal group) normalized sucrose preference and partially restored metabolic and inflammatory parameters. This study provided direct evidence that dietary interventions can correct obesity‐induced anhedonia, suggesting that both inflammatory and metabolic pathways contribute to hedonic deficits. These findings are in line with the previously reported beneficial effect of exercise.^38^

Given that the opioid system is relevant in nutrition, another study conducted an 8‐week randomized, double‐blind, placebo‐controlled “fast‐fail” proof‐of‐mechanism trial to assess the κ‐opioid receptor (KOR) antagonist JNJ‐67953964 (aticaprant) in 89 participants with clinically significant anhedonia.^85^ The primary outcome was ventral striatal activation during reward anticipation on fMRI (Monetary Incentive Delay task). The JNJ‐67953964 group (10 mg/day) showed significantly increased ventral striatum activation versus placebo and also improved on the SHAPS. There was also a reported correlation between changes in ventral striatal activation and SHAPS improvement. Although improvements on the Probabilistic Reward Task were less robust, the findings strongly supported KOR antagonism as a viable target mechanism for treating anhedonia and illustrated how neuroimaging endpoints can inform early‐stage antidepressant drug development. Results have been recently confirmed as adjunctive treatment to SSRI/SNRI in a Phase 2 randomized, double‐blind, placebo‐controlled study.^86^ Two recent separate lines of research further suggested further treatments targeting anhedonia. McIntyre et al.^87^ presented secondary and post‐hoc analyses of a Phase 3 randomized, double‐blind, placebo‐controlled trial of lumateperone 42 mg monotherapy in 376 patients with bipolar depression (both bipolar I and II disorders). Lumateperone broadly improved depressive symptoms across all MADRS items. Of particular relevance, the MADRS anhedonia factor improved significantly in the overall population, with even larger effects in the bipolar II subgroup, a traditionally difficult‐to‐treat disorder.

Hormonal status is important in depressive and other conditions. A randomized, double‐blind, placebo‐controlled trial investigated the role of hormone sensitivity in perimenopausal women receiving transdermal estradiol (TE2).^88^ Although the study focused on hormone sensitivity and anxiety‐related responses to E2 fluctuations, it also included the SHAPS to assess anhedonia. Baseline sensitivity of anxiety symptoms to endogenous estradiol predicted greater reward‐seeking behavior (measured by the Effort‐Expenditure for Rewards Task) in women treated with TE2. Although a direct anhedonia effect was not confirmed, the improved anxiety and indirectly improved reward motivation underscore the complex interplay between hormonal state, mood, and hedonic capacity.

A new antidepressant was tested in a Phase 3, double‐blind, placebo‐controlled trial examining ansofaxine (LY03005), a triple reuptake inhibitor, in 588 adults with MDD over 8 weeks.^89^ Patients received ansofaxine 80 mg/day, ansofaxine 160 mg/day, or placebo. Both ansofaxine doses showed robust improvement in MADRS total scores and achieved higher response and remission rates. Additionally, both doses significantly reduced anhedonia (MADRS anhedonia factor) while maintaining an acceptable safety profile.

On the other hand, trazodone is not new, but it is well known for its unique pharmacodynamic profile. A recent study conducted a 12‐week, open‐label, naturalistic trial comparing trazodone XR (150–300 mg/day) with SSRIs in 160 MDD patients.^90^ Trazodone XR displayed a relevant efficacy in reducing anhedonia scores (SHAPS) at Week 12, although the significance was affected by previous psychiatric treatment duration.

Finally, Velichkov et al.^91^ explored a non‐pharmacological, dietary intervention in a double‐blind, placebo‐controlled, randomized controlled trial in emerging adults (mean age = 20 years) with moderate‐to‐severe depressive symptoms. Participants (*n* = 60) received either daily blueberry supplementation or a placebo drink for 6 weeks. Acutely, at 2 h post‐ingestion of a single blueberry dose, positive affect and executive function (switch trial accuracy) improved significantly in the blueberry group, and these acute changes correlated with improved accuracy. However, at the 6‐week endpoint, the placebo group surprisingly showed greater reductions in depressive symptoms, anxiety, and anhedonia (SHAPS) than the blueberry group. The blueberry group's chronic intake did not produce lasting psychological improvements, and biomarker analyses (BDNF, IL‐6, hs‐CRP, oxidative stress markers) failed to detect treatment effects. Thus, while blueberries had an immediate positive impact, chronic supplementation may have induced a biphasic or hormetic‐like response that ultimately was less effective than placebo in improving mood and anhedonia.

Collectively, these lines of investigation underscore that anhedonia can be modulated by multiple therapeutic strategies: pharmacological agents (dopamine agonists, KOR antagonists, lumateperone, trazodone), hormonal interventions (TE2 in perimenopausal women), and lifestyle/dietary adjustments (food restriction in obesity, acute blueberry supplementation).

## DISCUSSION

**Figure 1:**
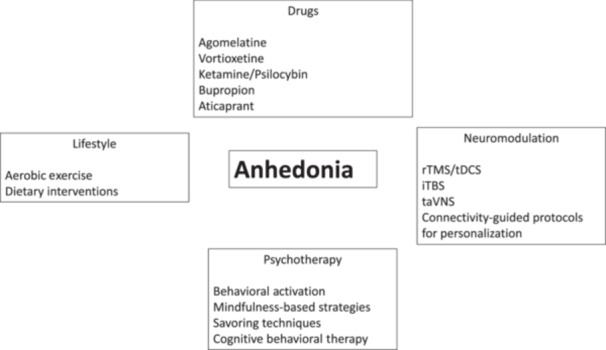
Anhedonia treatments. iTBS, intermittent theta‐burst stimulation; rTMS, repetitive transcranial magnetic stimulation; taVNS, transcutaneous auricular vagus nerve stimulation; tDCS; transcranial direct current stimulation.

The body of research reviewed, encompassing pharmacological interventions, psychotherapeutic and behavioral techniques, noninvasive neuromodulation strategies, and even dietary and lifestyle modifications, consistently underscores the possibility to treat deficits in pleasure, motivation, and reward responsiveness, though with a variable level of efficacy. Further details of the studies are reported in Table S1 and a summary of most relevant interventions is displayed in Figure 1 and Table 1.

**Table 1:** Summary table of promising anhedonia treatments in decreasing order of evidence.

Treatment	Evidence basis	Findings
Ketamine	Multiple RCTs, systematic review	Robust, rapid reduction in anhedonia for TRD/bipolar; effects last up to a week or more; linked to ACC/striatal network changes.
rTMS	Multiple RCTs	Significantly reduces anhedonia in treatment‐resistant depression. Targeting left DLPFC can improve reward sensitivity and reduce both anticipatory and consummatory deficits.
Vortioxetine	Multiple RCTs and pooled analyses	Consistent improvement in anhedonia across doses and durations; associated with better social functioning, quality of life, and dose‐dependent effects on hedonic capacity.
Agomelatine	Multiple studies, including RCTs	Rapid, sustained improvement in anhedonia; often superior to SSRIs/SNRIs for hedonic deficits. Generally fewer side‐effects, especially reduced emotional blunting.
Bupropion (±combos)	Several trials	Improves anhedonia and positive affect measures, especially useful adjunctively with SSRIs for partial responders. Acute emotional processing benefits observed, though immediate reward effects can be mixed.
Trazodone XR	One noninferiority trial	Outperformed SSRIs on SHAPS reduction and overall depressive symptom improvement. Offers better outcomes for insomnia and anxiety symptoms as well.
AXS‐05	One RCT versus bupropion	Combined dextromethorphan‐bupropion formulation showed faster, more robust antidepressant and anti‐anhedonic effects vs. bupropion alone.
Lumateperone	One RCT in bipolar depression	Significant improvement in MADRS anhedonia factor, especially pronounced in bipolar II. Demonstrated overall favorable safety/tolerability profile.
KOR antagonist	Single RCT	JNJ‐67953964 improved SHAPS and ventral striatum activation; well tolerated with moderate sample size (*n* = 89).
Psilocybin	Open‐label pilot	Rapid, enduring decreases in anhedonia for treatment‐resistant depression; small sample, but promising early‐phase evidence.
Behavioral/mindfulness‐based therapies	Multiple smaller RCTs	Generally reduces anhedonia. Effects often correlate with homework compliance (in BA) or enhanced mindfulness‐based reward responsiveness (in MORE). No single therapy clearly superior, but all show viability for anhedonia.

Abbreviations: ACC, anterior cingulate cortex; BA, behavioral activation; DLPFC, dorsolateral prefrontal cortex; KOR, κ‐opioid receptor; MADRS, montgomery‐asberg depression rating scale; MORE, mindfulness‐oriented recovery enhancement; RCTs, randomized control trials; rTMS, repetitive transcranial magnetic stimulation; SHAPS, Snaith–Hamilton Pleasure Scale; SNRIs, serotonin‐norepinephrine reuptake inhibitors; SSRIs, selective serotonin reuptake inhibitors; TRD, treatment‐resistant depression.

A central theme emerging from pharmacological research is that conventional antidepressants, while effective in reducing core depressive symptoms, often fail to fully restore the ability to experience pleasure and motivation.^14^, ^44^, ^92^ This gap has stimulated interest in agents and mechanisms that more directly target reward processing. For example, studies on agomelatine and brexpiprazole showed improvements in anhedonia and psychosocial functioning, especially when combined with exercise or as adjunctive therapies. Similarly, examinations of bupropion and venlafaxine suggested that enhancing dopaminergic and/or noradrenergic signaling can be particularly beneficial for patients with pronounced anhedonia. Mechanistic studies indicated that improvements in reward processing may be linked to biomarkers such as BDNF, reduced inflammation (e.g., CRP), and alterations in neural activity and functional connectivity within reward‐related brain circuits.

The potential of glutamatergic modulation via rapid‐acting treatments like ketamine or NMDA receptor modulators has also clearly emerged.^93^, ^94^, ^95^, ^96^, ^97^, ^98^, ^99^ Ketamine's rapid and robust effects on anhedonia—even before overall depressive symptoms remit—hint at mechanisms that bypass the slower neuroadaptive changes required by traditional monoaminergic antidepressants. Innovative interventions like psilocybin have suggested that resetting functional connectivity and introducing novel subjective experiences can reengage the brain's reward circuits, though it should still be considered an experimental treatment.^100^, ^101^ Likewise, novel pharmacological approaches, such as combining dextromethorphan‐bupropion (AXS‐05) or adjunctive brexpiprazole, highlight the potential synergy between multiple receptor targets and pathways.

Studies focusing on specific symptom clusters and subgroups have demonstrated that improvement in anhedonia correlates strongly with better psychosocial outcomes, including restored interest in previously enjoyable activities, improved quality of relationships, and enhanced daily functioning. For instance, studies with vortioxetine consistently show that reducing anhedonia mediates improvements in functioning and quality of life. Moreover, vortioxetine's effects appear to be dose‐dependent and long‐lasting, suggesting a durable anti‐anhedonic profile that can be maintained over extended periods. This finding is supported by analyses across diverse populations, including those with MDD and even individuals with remitted schizophrenia, where adding vortioxetine to antipsychotic therapy significantly reduced both physical and social anhedonia.

On the psychotherapeutic and behavioral side, a renewed focus on approaches such as BA, CBT, mindfulness‐based interventions, and savoring strategies aligns with the understanding that merely reducing negative affect is insufficient.^102^ Enhancing positive affect and reward responsiveness may be achieved through explicitly targeting engagement in enjoyable or meaningful activities, increasing attention to positive experiences, and training patients to anticipate and value future rewards. Studies directly comparing BA and MBCT for anhedonia found both approaches effective, though neither was clearly superior. The inclusion of savoring techniques or MORE can enhance the hedonic capacity, helping patients re‐learn to derive pleasure from daily events and natural rewards.

Interestingly, non‐pharmacological interventions, such as AE and dietary modifications (as seen with blueberry supplementation or weight‐management strategies), have also been explored, albeit with mixed or context‐dependent outcomes. Microbiome variability should probably be considered as a possible stratification factor.^103^, ^104^, ^105^

Neuromodulation techniques, including rTMS, tDCS, iTBS, and even taVNS, have increasingly been applied to address anhedonia. Studies indicate that targeting the DLPFC, DMPFC, or connecting these hubs to subcortical regions like the nucleus accumbens or ventral striatum can alter reward processing.

Despite these advances, several challenges remain. Many of the interventions discussed have shown promise in controlled trials, but questions about real‐world application, cost‐effectiveness, patient adherence, and long‐term maintenance persist.

Comorbidities and patient‐specific characteristics further complicate treatment selection. Co‐occurring marijuana use, older age of onset, or a family history of mental illness may predict less robust improvement in anhedonia. These findings underscore the need for comprehensive patient assessment and possibly adjunctive interventions (e.g., substance use treatment, family interventions) to maximize hedonic recovery.

An additional consideration is that while certain treatments (e.g., vortioxetine, behavioral activation, iTBS) show broad‐spectrum efficacy, others have a more specific effect: connectivity‐directed neuromodulation for anticipatory deficits, mindfulness or savoring‐based interventions for consummatory impairments, or dopaminergic‐enhancing medications for pervasive motivational blunting.

This narrative review is subject to several limitations. First, as a nonsystematic and narrative synthesis, it lacks the methodological rigor of predefined inclusion criteria, systematic search strategies, and formal risk‐of‐bias assessments associated with systematic reviews or meta‐analyses; however, multiple search criteria and cross‐referencing ensure that the relevant literature was included. Second, the selection of studies relied on the author's judgment of relevance and importance, potentially introducing selection bias and leaving out significant but less prominent findings. Third, heterogeneity in study designs, patient populations, outcome measures, and intervention protocols makes it difficult to draw firm, generalized conclusions about efficacy. In particular, many studies focused on short‐term outcome, while in clinical routine long‐term benefits are also important. However, the few studies that extended beyond the short term mainly supported a stable benefit.^37^, ^39^, ^62^, ^65^ Comorbid conditions, such as anxiety disorders or substance abuse, may also influence the clinical picture and outcome,^106^ therefore these factors should be evaluated in clinical practice. Finally, the review primarily covers recent literature, and there may be important older research not fully captured. Consequently, the conclusions should be interpreted with caution; however, the broad coverage may partially mitigate this limitation.

In conclusion, an increasing amount of evidence supports the need of effectively treating anhedonia as an integral step to achieving a fully meaningful recovery in depression and related disorders. The integration of these findings into routine clinical practice, coupled with ongoing research into mechanisms, biomarkers, and patient‐specific predictors, promises to achieve a personalized, dimension‐targeted intervention that restores not just the absence of negative mood, but the presence of pleasure, engagement, and a satisfying life.

## AUTHOR CONTRIBUTION

Author ideated, drafted and corrected the paper.

## CONFLICT OF INTEREST STATEMENT

Alessandro Serretti is or has been a consultant to or has received honoraria or grants unrelated to the present work from: Abbott, Abbvie, Angelini, Astra Zeneca, Clinical Data, Boheringer, Bristol Myers Squibb, Eli Lilly, GlaxoSmithKline, Innovapharma, Italfarmaco, Janssen, Lundbeck, Naurex, Pfizer, Polifarma, Sanofi, Servier, Taliaz. Alessandro Serretti is an Editorial Board member of *Psychiatry and Clinical Neurosciences Reports* and a co‐author of this article. To minimize bias, they were excluded from all editorial decision‐making related to the acceptance of this article for publication.

## ETHICS APPROVAL STATEMENT

N/A

## PATIENT CONSENT STATEMENT

N/A

## CLINICAL TRIAL REGISTRATION

N/A

## Supporting information

Supporting information.

## Data Availability

N/A
