# Supplementary material for: Anhedonia: Current and future treatments
Source: PCN Rep. 2025 Mar 23;4(1):e70088. doi: 10.1002/pcn5.70088 (PMC11930767; doi:10.1002/pcn5.70088)
Supplement: Supplementary file 1 — Supporting information. [file PCN5-4-e70088-s001.docx]

Supplementary table 1. Detailed studies description.

| Study | Objective | Design | Treatment | Subjects | Findings | Implications |
| --- | --- | --- | --- | --- | --- | --- |
| Alexander et al., 2023 | Ketamine on ACC connectivity | RCT, double-blind, crossover (~2 weeks) | Ketamine IV 0.5 mg/kg vs Placebo in TRD and HV (2-day post-infusion rsfMRI) | TRD=29, HV=21 | Ketamine improved MADRS (p=0.003) and TEPS (p<0.05), trend SHAPS (p=0.057). sgACC connectivity changes correlated with SHAPS, TEPS improvements. Enhanced sgACC-pgACC, sgACC-ventral striatum connectivity linked to reduced anhedonia. | Ketamine alters ACC connectivity patterns related to anhedonia improvement, underscoring ACC subregion roles in reward-related symptom changes. |
| Alsayednasser et al., 2022 | CBT/BA for anhedonia in COBRA | Secondary analysis of RCT (6–18 months follow-up) | CBT vs BA (up to 20 sessions each) | MDD=440 (CBT=219, BA=221) | SHAPS reduced by 5.70 points at 6 months (p<0.001) but no further improvement thereafter. Anhedonia remained elevated (SHAPS>25). Baseline anhedonia predicted poorer depression outcomes, especially in BA. Depression improved more than anhedonia. | Standard CBT/BA insufficiently improve anhedonia long-term. |
| Ballard et al., 2017 | Anhedonia & suicidal ideation | Post-hoc analysis (varied durations) | Ketamine IV 0.5 mg/kg in MDD/BD patients from 3 clinical trials | MDD=65, BD=35, total=100 | SHAPS associated with suicidal ideation at baseline (β=0.21,p=0.03) and changes in SHAPS correlated with SSI reduction (β=0.40,p=0.003) independent of depression severity. SHAPS explained 13% additional variance in suicidal ideation reduction. | Targeting anhedonia may reduce suicidal ideation independent of depressive symptoms, indicating ketamine’s anti-suicidal potential via reward mechanisms. |
| Bodén et al., 2021 | iTBS on anhedonia | RCT, double-blind, sham-controlled (10 weekdays) | iTBS (1200 pulses/session, twice daily, 90% RMT) vs Sham | Schizophrenia or depression=56 (Active=28, Sham=28) | CAINS total not significantly reduced overall (β=-3.46,95% CI:-7.45,0.53, p=0.088), but patients with depression showed significant reduction (β=-5.5, p=0.038). No significant effects in schizophrenia. iTBS well-tolerated. | iTBS over DMPFC may reduce anhedonia-related symptoms in depression but not in schizophrenia, supporting diagnosis-specific neuromodulation targets. |
| Bodkin et al., 1997 | Combining SRI+bupropion | Retrospective (mean 11 ± 14 months) | Bupropion (~243 ± 99 mg/day) plus SRI (~31 ± 16 mg/day fluoxetine-equivalent) | Affective disorders=27 partial/unsatisfactory responders | 70% (19/27) showed greater improvement with combination than monotherapy. No seizures reported. Few adverse effects. | Adding bupropion to SRI may enhance symptom control in partial responders with tolerable adverse effects, offering a strategy for TRD. |
| Bogaert et al., 2023 | FEST on anhedonia & dampening | RCT (~2 weeks intervention, follow-up at 2 weeks) | FEST vs Control (2×90-min online sessions) | Undergraduates=155 (FEST=75, Control=80) | FEST improved episodic future thinking metrics (p<0.001) but no significant changes in anhedonia (p>0.05) or dampening (p>0.05). No effects on depressive symptoms or optimism/pessimism. Mild symptom levels at baseline. | FEST enhances future thinking but does not reduce anhedonia under these conditions; adaptations may be needed for clinically anhedonic populations. |
| Boyer et al., 2000 | Sequential MDD symptom improvement | Open-label (8 weeks) | Sertraline 50–150 mg/day | MDD=140 ITT | Anxiety improved first (Days 0–7), then depression (Days 7–21), then anhedonia (Days 21–56). HAM-D from 28.06±6.0 to 7.63±5.2(p<0.01). | Sertraline’s effects unfold in a sequence: anxiety→depression→anhedonia improvement. Guides expectations and management of symptom progression in MDD. |
| Cao et al., 2019 | Vortioxetine on anhedonia in MDD | Post-hoc (8 weeks, open-label) | Vortioxetine 10–20 mg/day | MDD=95 | SHAPS:6.2±3.8→3.3±3.8 (p<0.0001); MADRS anhedonia factor:18.3±3.7→11.4±6.1 (p<0.0001). Anhedonia improvements correlated with better social functioning (SDS) and quality of life (WHO-5). Predictors of non-remission: marijuana use, older onset, family history. | Vortioxetine significantly improves anhedonia and related functioning in MDD. |
| Carhart-Harris et al., 2016 | Psilocybin on TRD | Open-label (2 sessions: Day 0=10 mg, Day 7=25 mg, follow-up 3 months) | Psilocybin oral 10 mg then 25 mg one week apart + psychological support | TRD=12, mean illness duration=17.8±8 yrs | QIDS:19.2±2.0→7.4±4.9 at 1 wk (p=0.002); SHAPS:7.5±3.7→1.4±2.7 at 1 wk (p=0.002); sustained improvement up to 3 months. Mild transient adverse events only. | Psilocybin yields rapid, enduring reductions in depression and anhedonia in TRD. |
| Cernasov et al., 2023 | Homework dynamics in CBT | RCT comparing BATA & MBCT (up to 15 sessions) | BATA vs MBCT; Homework completion measured Pt-Hw & Cl-Hw | Anhedonia=73 (BATA=38, MBCT=35; final n=51 completers) | Both treatments improved SHAPS. Session-to-session Pt-Hw completion (p=0.003) and Cl-Hw in BATA (p=0.003) correlated with greater SHAPS improvement. | Within-person homework variability matters more than overall completion, especially in BATA, for improving anhedonia. |
| Cernasov et al., 2024 | BATA vs MBCT for anhedonia | RCT (8–15 sessions) | BATA vs MBCT | Anhedonia=116 (BATA=61, MBCT=55; SHAPS≥20) | SHAPS decreased in both groups (BATA:-7.20, MBCT:-7.00, p<0.001), no between-group difference (Δ=-0.20,p=0.845,d=0.05). Both reduced internalizing symptoms. MBCT had higher attrition trend. | Both BATA and MBCT reduce anhedonia without one clearly superior. Both are viable clinical options. |
| Corruble et al., 2013 | Agomelatine vs Escitalopram | RCT, double-blind (24 weeks) | Agomelatine 25–50 mg/d vs Escitalopram 10–20 mg/d | MDD=324 (Ago=164, Esc=160) | Both improved HAMD-17 scores. At 24 weeks, remission ~69.6% Ago vs 63.1% Esc. Agomelatine improved sleep quality in severe sleep complaints (p=0.016, p=0.009) and caused less emotional blunting (28% vs 60%, p=0.024). | Agomelatine equals escitalopram’s efficacy but offers better sleep, less emotional blunting. |
| Dalhuisen et al., 2024 | rTMS vs med switch in TRD | RCT, multicenter, (8 weeks) | rTMS (25 high-freq sessions left DLPFC) vs Antidepressant switch+psychotherapy | TRD=89 (MDD nonpsychotic) | rTMS>med switch: HAM-D reduction 11.2 vs 6.3(p<0.01). Response: rTMS=37.5%, switch=14.6%; Remission: rTMS=27.1%, switch=4.9%. Greater improvement in anhedonia & anxiety with rTMS. | rTMS outperforms medication switching in TRD, significantly aiding anhedonia. |
| De Berardis et al., 2017 | Agomelatine on CRP, anhedonia | Open-label (12 weeks) | Agomelatine 25–50 mg/day | MDD=30 | HAM-D: 28.2±2.8→9.7±4.9 (p<0.001), SHAPS:6.6±2.2→3.1±2.0 (p<0.001), CRP:2.5±0.6→1.8±0.5 mg/L (p<0.001), CRP↓ only in remitters; no serious AEs | Agomelatine reduces depression, anhedonia, inflammation; remission links to CRP↓ |
| Di Giannantonio et al., 2011 | Agomelatine on anhedonia | Open-label (8 weeks) | Agomelatine 25–50 mg/day | MDD=30; completed=24(80%) | HAM-D (p<0.05), HAM-A (p<0.01), SHAPS (p<0.05) improved; week 1 responders=9(30%), remission end=18(60%); no serious AEs | Early improvement in depression, anxiety, anhedonia, safe profile |
| Di Giannantonio et al., 2019 | Agomelatine pooled analysis | Pooled open-label (up to 12 weeks) | Agomelatine 25–50 mg/day | MDD=1942 | svMADRS:34.2→10.7 (p<0.001), response=79.2%, remission=63.9%; SHAPS:11.3→1.1 (p<0.001), 84.8% anhedonia remission; no serious AEs | Anhedonia improvement relevant for overall depression recovery. |
| Dudek et al., 2023 | Trazodone XR vs SSRIs | Open-label, non-inferiority (12 weeks) | Trazodone XR (150–300 mg/day) vs SSRIs (various doses) | MDD=160 (TRZ=79, SSRIs=81) | MADRS at Week 12: TRZ=5.99 vs SSRIs=11.06 (p=0.001). QIDS-CR remission: TRZ=78.7% vs SSRIs=52.2% (p=0.003). SHAPS at Week 12: TRZ=2.56 vs SSRIs=4.43(p=0.011). Better insomnia, anxiety outcomes with TRZ. | Trazodone XR outperformed SSRIs in depressive, anhedonic, anxiety, and insomnia measures. |
| Farabaugh et al., 2015 | CBT vs Escitalopram cognitions | RCT (12 weeks) | CBT vs Escitalopram (10–20 mg/day) | MDD=26 (CBT=15, Esc=11) | Both treatments significantly improved depression, anhedonia (SHAPS), cognitive measures, quality of life. No significant between-group differences. | CBT and escitalopram yield similar improvements in mood, anhedonia, and cognitions, suggesting convergent mechanisms of change in MDD treatment. |
| Ferstl et al., 2024 | taVNS on invigoration | Single-blind crossover (~2 sessions) | taVNS (cymba conchae) vs Sham (earlobe) | MDD=30, HC=29 | taVNS increased invigoration (p=0.040, d=0.55) and wanting in MDD on Session 1, sustained Session 2. | taVNS enhances effort and reward wanting in MDD. |
| Fukuda et al., 2021 | rTMS in TRD anhedonia | Retrospective (~35 sessions) | Left DLPFC rTMS (10 Hz,120% MT, some 5 Hz due to tolerability) | TRD=144 | SHAPS:8.10→3.06 (p<0.001, d=1.45), 58.55% reduction. Depression (IDS-SR) also improved. Baseline anhedonia did not affect response. | rTMS robustly reduces anhedonia in TRD regardless of baseline severity. |
| Gargoloff et al., 2016 | Agomelatine in real-world anhedonia | Open-label, multicenter (8 weeks) | Agomelatine 25–50 mg/day | MDE=257 enrolled, analyzed=143 | SHAPS:8.5→4.1 (p<0.001) by week 8, improvement from week 1 (p<0.01); QIDS-SR-16, GAD-7 improved (p<0.001); correlations with anhedonia; ADR=10.5% | Rapid anhedonia improvement in routine clinical practice |
| Garland et al., 2021 | MORE in chronic opioid users | RCT (8 weeks + 4-month follow-up) | MORE vs Supportive Group (SG) | Veterans on LTOT=63 (MORE=32, SG=31) | MORE reduced SHAPS more than SG (p=0.015), normalizing anhedonia by follow-up. LPP and SCL responses to natural rewards increased, correlating with anhedonia improvement (r=-0.34,p=0.01). | Mindfulness-based intervention (MORE) restores hedonic capacity and reward responses in chronic opioid users. |
| Garland et al., 2024 | MORE in Veterans with chronic pain | RCT, single-blind (8 weeks) | MORE vs Supportive Psychotherapy (SP) | Chronic pain on LTOT=230 (MORE=116, SP=114) | MORE reduced anhedonia (SHAPS LSMD=-2.27, p<0.001), pain, opioid dose, craving, and improved positive affect. Better than SP for multiple outcomes. | MORE offers effective treatment for chronic pain, opioid misuse, and anhedonia in Veterans. |
| Greš et al., 2024 | Vortioxetine in schizophrenia anhedonia | RCT (12 weeks) | Vortioxetine 10 mg/day + antipsychotic vs Antipsychotic alone | Schizophrenia (remitted)=120 (Intervention=60,  Control=60) | Physical anhedonia (CPAS): Intervention:47.6→41.6 vs Control:48.4→48.4 (p<.001,η²=0.733). Social anhedonia (CSAS): Intervention:28.5→24.2 vs Control:28.4→28.6 (p<.001,η²=0.618). Greatest improvement with vortioxetine+olanzapine. | Adding vortioxetine reduces physical/social anhedonia in remitted schizophrenia, especially with olanzapine. |
| Grillo et al., 2014 | Dietary restriction effect | Animal study, longitudinal (~40 days) | Food restriction vs ad lib in hypo-IRAS rats, Control group | Rats: hypo-IRAS vs prevention/reversal groups vs control | Obesity-induced anhedonia reversed by food restriction. Sucrose preference restored, leptin/triglycerides/BDNF/inflammatory markers normalized (p<0.05). | Metabolic and inflammatory factors contribute to obesity-related anhedonia; dietary restriction can restore hedonic function. |
| Hanuka et al., 2023 | iCBT on anhedonia & reward | RCT (10 weeks) | iCBT vs monitored attention control (MAC), plus Healthy Controls (HC) | MDD=52 (iCBT=26, MAC=26), HC=42 | iCBT reduced SHAPS (p=0.001) more than MAC (p=0.006). iCBT enhanced Nacc and sgACC activation. Nacc changes mediated anhedonia reduction. HC showed no change. | iCBT decreases anhedonia by enhancing reward circuit function. |
| Katzman et al., 2022 | Adjunctive brexpiprazole | Post hoc of 4 RCTs (6 weeks) | Brexpiprazole 2 mg or 2–3 mg vs Placebo | MDD with inadequate AD response: 2 mg=486, 2–3 mg=770, PLC(2 mg)=585, PLC(2–3 mg)=788 | Significant improvement in all MADRS symptom clusters, largest effect on anhedonia (2 mg: p<0.0001, d=0.43). Early, sustained improvements noted. | Brexpiprazole targets core MDD symptoms including anhedonia. |
| Kazour et al., 2023 | Olfactory hedonic changes | Open-label (8 weeks) | Escitalopram (dose not specified) | MDE=52 (43 completed; Responders=24, Nonresponders=19) | Only responders showed significant improvement in hedonic perception of pleasant odors (p=0.018), no change in unpleasant odor ratings or olfactory threshold/identification. | Improvements in hedonic odor perception may reflect antidepressant response independent of olfactory sensitivity, potential sensory biomarker of treatment efficacy. |
| Kong et al., 2024 | tDCS on anhedonia | RCT, double-blind, sham-controlled (4 weeks) | DLPFC anodal, OFC cathodal, or Sham (12×20-min sessions) | Anhedonic subjects=70 | SHAPS reduced most in DLPFC vs sham at 2 weeks (p=0.028), 80.83% reduction at 4 weeks, 85.99% at 8 weeks (p=0.066). OFC less effective for anhedonia but improved QIDS-SR16. DLPFC superior in anhedonia improvement. | Anodal tDCS over left DLPFC offers sustained anhedonia improvement in depression. |
| Kos et al., 2024 | iTBS/tDCS in schizophrenia apathy | Multicenter RCT, double-blind (~2 weeks) | iTBS or tDCS to right DLPFC vs Sham | Schizophrenia/apathy=88(Active iTBS=32, Sham iTBS=16, Active tDCS=17, Sham tDCS=17) | No significant difference vs sham for apathy (AES-C) or negative symptoms. Minor overall improvements likely placebo/participation effect. No serious AEs. | iTBS/tDCS did not outperform sham for apathy in schizophrenia, suggesting current parameters insufficient or requiring different protocols. |
| Krystal et al., 2020 | KOR antagonist | RCT, double-blind, placebo-controlled (8 weeks) | JNJ-67953964 10 mg/day vs Placebo | Anhedonia+Mood/Anxiety=89 (JNJ=45, PLC=44) | Ventral striatum activation↑ vs placebo (p<0.01; Hedges’ g=0.58). SHAPS improved (p=0.0345; g=0.44). Overall treatment effect on PRT (p=0.03; g=0.49). Well-tolerated. | Supports KOR antagonism’s neural and clinical anti-anhedonic potential. |
| Kumar et al., 2024 | BA+S vs EA on positive affect | RCT (21 days follow-up) | BA+S: 2×60-min virtual sessions, EA: 2×60-min virtual sessions | Low positive affect university students=60 (BA+S=30, EA=30) | BA+S increased daily positive affect (p=0.01) vs no change in EA. Positive valence improved more in BA+S (p<0.001, d=0.90). BA+S also superior in improving negative valence symptoms (p=0.035, d=0.33). High satisfaction and EMA compliance. | BA+S is a brief, scalable intervention enhancing positive affect and reducing distress in students. |
| Lally et al., 2014 | Ketamine’s anti-anhedonia | RCT, double-blind, crossover (10–14 days) | Ketamine IV 0.5 mg/kg vs Placebo (0.9% saline) separated by 2-week washout | Bipolar depression=36 (BPI/II) | Ketamine significantly reduced SHAPS scores (p<0.001) within 40 min up to 14 days, independent of MADRS; increased dACC, putamen metabolism correlating with anti-anhedonic effects; lithium users showed greater benefit (p=0.06). | Ketamine rapidly improves anhedonia in TR bipolar depression, linked to dACC/putamen metabolism. |
| Lally et al., 2015 | Ketamine & anhedonia changes | Open-label (single infusion, up to 28 days) | Ketamine IV 0.5 mg/kg single infusion | TR MDD=52; FDG-PET subset=20 | SHAPS ↓6.45 points at 40 min (p<0.001), lasting ≥3 days. Hippocampus/dACC metabolism ↑, OFC metabolism ↓ associated with anti-anhedonic effects. Riluzole did not enhance effects. | Single ketamine infusion reduces anhedonia rapidly, correlating with specific metabolic changes in hippocampus/dACC/OFC. |
| Light et al., 2011 | RVLPFC, positive affect, AD treatment | RCT, double-blind (8 weeks, follow-ups at 6 months) | Antidepressant: venlafaxine-ER or fluoxetine vs Control | MDD=27 (medication-free), HC=19 | Lower RVLPFC activity during positive affect suppression predicted greater reduction in anhedonia at 8 weeks (p<0.05), explaining 61% variance. | Identifies RVLPFC activity as a neural predictor of anhedonia improvement, informing treatment stratification and understanding of recovery processes. |
| Light et al., 2019 | rTMS on subtle positive cues | RCT, double-blind (4 weeks, 20 sessions) | Active rTMS (10 Hz,3000 pulses/session,120% MT) vs Sham over left DLPFC | MDD=19 baseline MADRS≥18 | Improved accuracy for low-intensity happy faces in active rTMS (p<0.05), correlated with SHAPS reduction. SHAPS improved (p<0.05). Empathic happiness changes predicted reduced anhedonia. High-intensity faces no change. | rTMS enhances sensitivity to subtle positive stimuli, reducing anhedonia and linking empathic happiness changes to hedonic improvement. |
| Martinotti et al., 2012 | AG vs VLX anhedonia | Open-label, parallel (8 weeks) | Agomelatine 25–50 mg/day vs Venlafaxine XR 75–150 mg/day | MDD=60 (AGO=30,VLX=30) | SHAPS reduced more with AGO (p<0.001) than VLX (p<0.05), evident by week 1 (p<0.05). HAM-D, HAM-A improved similarly. AGO improved CGI (p<0.05), VLX did not (p=0.18). Fewer AEs with AGO (3.2%) vs VLX (39.2%). | Agomelatine provides quicker, greater anhedonia relief with better tolerability than venlafaxine |
| Martinotti et al., 2016 | Agomelatine, BDNF, anhedonia | Open-label (8 weeks) | Agomelatine 25–50 mg/day | Depressive disorders=27 | BDNF:117.35±34.02→150.79±42.78 ng/mL (p<.01), SHAPS:5.82→3.11 (p<.01), BDNF changes correlate with improved depression/anhedonia; mild AEs only | BDNF increase may signal response; agomelatine improves mood and anhedonia |
| Mattingly et al., 2023 | Long-term vortioxetine | Two 52-week open-label extensions after DB trials | Vortioxetine flexible-dose (5–10 mg/day or 15–20 mg/day) | MDD= Study 1: N=74, Study 2: N=71 | MADRS total improvements maintained; at Week 52: 5–10 mg group: -4.3±9.2 points, 15–20 mg: -10.9±10.0 points. Anhedonia factor improved (5–10 mg: -3.10±0.57, 15–20 mg: -5.62±0.60). Vortioxetine well-tolerated. | Vortioxetine shows sustained efficacy and safety, with marked long-term anti-anhedonic effects in MDD. |
| McCabe et al., 2010 | SSRI vs NRI neural effects | RCT, double-blind (7 days) | Citalopram 20 mg/day vs Reboxetine 4 mg twice daily vs Placebo | Healthy=45 (15 each group) | Citalopram reduced ventral striatum/orbitofrontal BOLD response to chocolate (p=0.001), reboxetine enhanced medial OFC response (p=0.05). No subjective changes. | SSRIs may cause emotional blunting by reducing reward/aversion neural responses, whereas NRIs preserve/enhance reward-related activity. |
| McIntyre et al., 2016 | Levomilnacipran on MDD symptoms | Post hoc of 5 RCTs (8–10 weeks) | Levomilnacipran ER vs Placebo | MDD=2,598 (Levo=1,566, PLC=1,032) | Significantly improved MADRS items. Anhedonia improved (OR=1.65,p<0.0001). Early onset (Week 1) and broad symptom coverage. | Levomilnacipran ER effectively reduces anhedonia and broad depressive symptoms, providing early and extensive symptom relief in MDD. |
| McIntyre et al., 2021 | Vortioxetine pooled analysis in anhedonia | Post-hoc of 11 short-term RCTs (6–8 weeks) | Vortioxetine 5–20 mg/day vs Placebo | MDD= Vortioxetine=3,219, Placebo=1,769 | Dose-dependent anhedonia improvement (e.g., 20 mg: -2.24 vs placebo, p<0.001). Functioning (SDS) improved via anhedonia reduction. | Vortioxetine’s anti-anhedonic effects drive functional gains, dose dependent. |
| McIntyre et al., 2023 | Lumateperone in bipolar | RCT, double-blind, placebo-controlled (6 weeks) | Lumateperone 42 mg vs Placebo | Bipolar depression=376 (BPI=300, BPII=76) | MADRS LSMD=-4.6 (p<0.0001) overall. Anhedonia factor improved (LSMD=-2.4, p<0.0001). Bipolar II showed greater improvement in anhedonia (LSMD=-4.5, p<0.001). Broad symptom improvement, good safety. | Lumateperone improves depressive and anhedonic symptoms in bipolar disorder, more in bipolar II. |
| McIntyre et al., 2024 | Venlafaxine on anhedonia | Post hoc pooled analysis (8 weeks) | Venlafaxine XR vs Placebo | MDD=1,087 (Venla=585, PLC=502) | Venlafaxine XR reduced MADRS anhedonia subscale more than placebo (difference=-2.73, p<0.0001), significant from Week 2. Greater baseline severity = greater improvement. Also improved amotivation. | Venlafaxine XR improves anhedonia and amotivation early in treatment, more beneficial for patients with pronounced motivational deficits. |
| Mi et al., 2023 | Ansofaxine efficacy in MDD | RCT, double-blind, placebo-controlled (8 weeks) | Ansofaxine 80 mg/day vs Ansofaxine 160 mg/day vs Placebo | MDD=588 (80 mg=187,160 mg=186, PLC=185) | MADRS difference vs placebo:80 mg=-5.46, p<0.0001;160 mg=-5.06,p <0.0001. SHAPS improvement 80 mg: -1.58, p<0.0001;160 mg:-1.60, p<0.0001. Response~79.89%, Remission≥51.63%. Mild/moderate AEs. | Ansofaxine significantly improves depressive and anhedonic symptoms with favorable tolerability. |
| Nogo et al., 2022 | Ketamine on anhedonia | Systematic review (preclinical & clinical) | Ketamine various doses (mainly 0.5 mg/kg IV) acute/chronic | 30 studies (11 clinical,19 preclinical) | Ketamine reduced anhedonia across anticipatory, consummatory, and motivation-related domains. Effects associated with BDNF, glutamate, dopaminergic changes, brain regions (ventral striatum, PFC), lasting up to 7 days. | Ketamine robustly improves multidimensional anhedonia via neuroplastic and reward pathway modulation. |
| Pettorruso et al., 2018 | rTMS in cocaine use | Pilot open-label (5 days, twice daily) | rTMS (15 Hz, 2400 pulses/session, 100% MT) over left DLPFC | Cocaine use disorder=15 | TEPS anticipatory pleasure ↑17.47% (p<0.01), consummatory ↑12.45% (p<0.01), anhedonia (CSSA)↓ (p<0.01), craving↓ 51.45% (p<0.01), 71.4% UDS negativity. Negative correlation between craving reduction & anhedonia improvement (p=0.002). | High-frequency rTMS improves anhedonia and reduces craving in CUD. |
| Potsch & Rief, 2024 | BA/MG/COM for reward & depression | RCT (2 weeks) | BA vs MG vs COM vs WL (2-week online interventions) | N=336 (per-protocol=224), mild depression | PHQ-9: BA,MG,COM >WL (p<0.05). PVSS-21 reward sensitivity: BA,MG>WL, COM not significant. SHAPS anhedonia: all active>WL (p<0.001). No difference among active groups. Brief interventions improved depression, reward, anhedonia. | Short online BA or MG interventions enhance reward sensitivity and reduce anhedonia, though combining them provides no additional benefit. |
| Sun et al., 2023 | AG+AE vs AG effects | RCT (12 weeks) | Agomelatine 25 mg/day (↑50 mg/day at week 2) ± AE (45-min, 3×/week) | MSD=178; AG=90, AG+AE=88 | Greater reductions in HAM-D, BDI, SHAPS, CRP with AG+AE vs AG alone (p<0.05); AG+AE remission=34 (38.64%) vs AG=27 (30%), p=0.008; fewer adverse events | AG+AE synergy enhances antidepressant, anti-anhedonic, anti-inflammatory effects |
| Swales et al., 2023 | Hormone sensitivity TE2 | RCT, double-blind, placebo-controlled (8 weeks) | Transdermal estradiol 0.1 mg/24h vs Placebo | Perimenopausal women=66 (TE2=32, PLC=34) | E2-anxiety sensitivity predicted higher EEfRT reward-seeking under TE2 (p<0.001), moderated by recent SLEs. E2-anhedonia sensitivity was not predictive (p=0.935). Anxiety/anhedonia improved overall in TE2 group. | Baseline hormone sensitivity and stress exposure guide TE2’s reward benefits, informing individualized treatment approaches. |
| Tabuteau et al., 2022 | AXS-05 vs bupropion | RCT, double-blind (6 weeks) | AXS-05 (Dextromethorphan-Bupropion 45 mg/105 mg once daily 3 days, then twice daily 6 weeks) vs Bupropion SR 105 mg tablet same schedule | MDD=97 randomized (AXS-05=48, Bupropion=49; efficacy population=80) | AXS-05 showed greater MADRS reduction over Weeks 1–6 than bupropion (Difference=-4.9, p<0.001) and higher remission at Week 6 (46.5% vs 16.2%, p=0.004). Core symptoms (MADRS-6) and CGI-S improved more with AXS-05. Mild-to-moderate AEs, no severe effects. | AXS-05 provides faster, more robust antidepressant effects than bupropion, improving remission and core symptoms. |
| Tan et al., 2021 | Vortioxetine in MCI cognition | Open-label (6 months) | Vortioxetine 5 mg/day (up-titration to 10 mg/day if needed) | MCI=111 enrolled,83 analyzed (PHQ-9 ≤4) | MoCA:24.2±1.7→29.7±0.9 (p<0.001). DSST improved (33.0±13.0→45.5±14.1, p<0.001). CDR memory domain improved, 89.6% showed overall improvement (CIBIC+). No severe AEs. | Vortioxetine enhances cognition in MCI, showing promise as a cognitive enhancer with good tolerability. |
| Tomarken et al., 2004 | Bupropion on anhedonia | RCT, double-blind (12 weeks total: Phase 1=6 weeks, Phase 2=6 weeks) | Bupropion SR: Phase 1=300 mg/day, Phase 2=400 mg/day; Placebo: Phase 1 placebo, switched to Bupropion SR 300 mg/day in Phase 2 | MDD=19 (Bupropion n=10, Placebo n=9) | Bupropion SR produced a significant linear decline in MASQ Anhedonic Depression (p=0.02) versus placebo. Positively keyed items improved with bupropion (p=0.0002). No significant between-group differences in anxiety measures; placebo affected general distress but not anhedonia. | Bupropion SR preferentially improves anhedonia, potentially useful for patients with pronounced hedonic deficits. |
| Velichkov et al., 2024 | Biphasic blueberry effects | RCT, double-blind, placebo-controlled (6 weeks) | Blueberry drink (22 g powder/day) vs Placebo | Moderate-severe depression =60 | Acute: Positive affect↑ (p=0.026), exec. function↑(p=0.025). Chronic: BDI-II, SHAPS, GAD-7 improved more in placebo (p=0.023 for BDI-II, p=0.029 SHAPS). No biomarker changes. | Suggests acute benefits but diminished chronic effects, indicating a biphasic or hormetic response to blueberry supplementation. |
| Vinckier et al., 2017 | Anhedonia predicts functioning | Cohort (10–14 weeks) | Agomelatine | MDD=1570 outpatients | Improvement in anhedonia predicts psychosocial improvement, OR=7.3 (95% CI:4.3–12.1, p<0.0001); mediation shows anhedonia as key; persistent anhedonia→poor function | Addressing anhedonia essential for functional recovery |
| Walsh et al., 2018 | Acute bupropion effects | RCT, double-blind (single dose, testing at 3 hours) | Bupropion SR 150 mg single dose vs. Placebo | Healthy=40 (Bupropion n=20, Placebo n=20) | Bupropion acutely improved recognition of happy faces (p<0.05) and reduced negative bias, but did not enhance reward processing. Instead, it decreased selecting high-probability wins (p<0.01) and slightly increased SHAPS scores (p=0.06), suggesting dissociation between emotional and reward effects. | Bupropion’s acute emotional processing benefits contrast with no immediate reward processing gains, indicating distinct underlying mechanisms. |
| Wang et al., 2021 | Connectivity rTMS | RCT, double-blind, sham-controlled (15 days) | rTMS (10 Hz,100% MT) targeting lDLPFC-NAcc connectivity vs Sham | Depression=56 (Real=32, Sham=24) | TEPS-anti improved in Real (p=0.001,d=0.618), no change in Sham. HAMD response: Real=65%,Sham=25% (p=0.003). ERP measures (cue-P3) correlated with TEPS-anti improvement. Early and strong effect on anticipatory anhedonia. | Connectivity-directed rTMS enhances reward anticipation and anhedonia in depression, with cue-P3 as a potential biomarker of efficacy. |
| Watanabe et al., 2022 | Vortioxetine in Japanese MDD | Post-hoc (8-week, DB, placebo-controlled) | Vortioxetine 10 mg vs 20 mg vs Placebo | MDD=489 (Placebo=161,10 mg=165,20 mg=163) | MADRS anhedonia factor reduced more with vortioxetine (10 mg diff=-1.34,p=0.03; 20 mg diff=-1.77,p=0.004), greater impact at 20 mg. Effects more pronounced with baseline anhedonia≥18. | Vortioxetine improves anhedonia in Japanese MDD, with 20 mg offering stronger benefits and greater efficacy in severe anhedonia. |

Legend: ADTs: Antidepressant Treatments; AE: Aerobic Exercise; AEs: Adverse Events; AG: Agomelatine; AMPS: Enjoyment of small pleasures measure; BA: Behavioral Activation; BA+S: Behavioral Activation plus Savoring; BATA: Behavioral Activation Treatment for Anhedonia; BD: Bipolar Disorder; BDI-II: Beck Depression Inventory-II; BDNF: Brain-Derived Neurotrophic Factor; BPI: Bipolar I; BPI/II: Bipolar I or II Disorder; BPII: Bipolar II; BPRS: Brief Psychiatric Rating Scale; CAINS: Clinical Assessment Interview for Negative Symptoms; CDR: Clinical Dementia Rating; CDSS: Calgary Depression Scale for Schizophrenia; CGI-S: Clinical Global Impression - Severity; CGI: Clinical Global Impression; CI: Confidence Interval; CIBIC+: Clinician Interview-Based Impression of Change Plus; COBRA: Cost and outcome of behavioural activation versus cognitive behaviour therapy for depression study; COM: Combination group; CPAS: Chapman Scale for Physical Anhedonia; CRP: C-Reactive Protein; CSAS: Chapman Scale for Social Anhedonia; CUD: Cocaine Use Disorder; dACC: dorsal Anterior Cingulate Cortex; DASS: Depression, Anxiety, and Stress Scale; DB: Double-Blind; DLPFC: Dorsolateral Prefrontal Cortex; DSST: Digit Symbol Substitution Test; EA: Emotional Awareness; EEfRT: Effort-Expenditure for Rewards Task; EFT: Episodic Future Thinking; ERP: Event-Related Potential; Esc: Escitalopram; FDG-PET: Fluorodeoxyglucose Positron Emission Tomography; FEST: Future Event Specificity Training; GAD-7: Generalized Anxiety Disorder 7-item scale; GAF: Global Assessment of Functioning; HAM-A: Hamilton Anxiety Rating Scale; HAM-D: Hamilton Depression Rating Scale; HC: Healthy Controls; hs-CRP: high-sensitivity C-Reactive Protein; IL-6: Interleukin-6; iTBS: Intermittent Theta-Burst Stimulation; IV: Intravenous; JNJ: JNJ-67953964; KOR: κ-Opioid Receptor; Levo: Levomilnacipran; LPP: Late Positive Potential; LSEQ: Leeds Sleep Evaluation Questionnaire; LSMD: Least Squares Mean Difference; LTOT: Long-Term Opioid Therapy; MAC: Monitored Attention Control; MADRS: Montgomery-Åsberg Depression Rating Scale; MASQ: Mood and Anxiety Symptom Questionnaire; MBCT: Mindfulness-Based Cognitive Therapy; MDD: Major Depressive Disorder; MG: Mindfulness and Gratitude; MoCA: Montreal Cognitive Assessment; MORE: Mindfulness-Oriented Recovery Enhancement; MSD: Moderate to Severe Depression; MT: Motor Threshold; Nacc: Nucleus Accumbens; NRI: Noradrenergic Reuptake Inhibitor; OFC: Orbitofrontal Cortex; PANAS: Positive and Negative Affect Schedule; PCL-5: PTSD Checklist for DSM-5; pgACC: perigenual Anterior Cingulate Cortex; PHQ-9: Patient Health Questionnaire-9; PLC: Placebo; PSQI: Pittsburgh Sleep Quality Index; PSS: Perceived Stress Scale; PSWQ: Penn State Worry Questionnaire; PVSS-21: Positive Valence System Scale-21; Q-LES-Q: Quality of Life Enjoyment and Satisfaction Questionnaire; QIDS-SR-16: Quick Inventory of Depressive Symptomatology, 16-item Self-Report; QIDS: Quick Inventory of Depressive Symptoms; RCT: Randomized Controlled Trial; rsfMRI: resting-state Functional Magnetic Resonance Imaging; rTMS: Repetitive Transcranial Magnetic Stimulation; RVLPFC: Right Ventrolateral Prefrontal Cortex; S: Savoring; SANS: Scale for the Assessment of Negative Symptoms; SCL: Skin Conductance Level; SDS: Sheehan Disability Scale; sgACC: subgenual Anterior Cingulate Cortex; SHAPS: Snaith-Hamilton Pleasure Scale; SLEs: Stressful Life Events; SOD: Superoxide Dismutase; SR: Sustained Release; SSI: Scale for Suicide Ideation; SSRI: Selective Serotonin Reuptake Inhibitor; svMADRS: shortened version of Montgomery-Åsberg Depression Rating Scale; taVNS: Transcutaneous Auricular Vagus Nerve Stimulation; TBARS: Thiobarbituric Acid Reactive Substances; tDCS: Transcranial Direct Current Stimulation; TE2: Transdermal Estradiol; TEPS: Temporal Experience of Pleasure Scale; TRD: Treatment-Resistant Depression; TRZ: Trazodone; Venla: Venlafaxine; WHO-5: World Health Organization-5 Well-Being Index; WL: Waitlist control; XR: Extended Release.
